# Techno-Functional, Nutritional and Environmental Performance of Protein Isolates from Blue Lupin and White Lupin

**DOI:** 10.3390/foods9020230

**Published:** 2020-02-21

**Authors:** Martin Vogelsang-O’Dwyer, Juergen Bez, Iben Lykke Petersen, Marcel Skejovic Joehnke, Andreas Detzel, Mirjam Busch, Martina Krueger, Lilit Ispiryan, James A. O’Mahony, Elke K. Arendt, Emanuele Zannini

**Affiliations:** 1 School of Food and Nutritional Sciences, University College Cork, Cork T12 K8AF, Ireland; m.vogelsangodwyer@umail.ucc.ie (M.V.-O.); lilit.ispiryan@umail.ucc.ie (L.I.); e.zannini@ucc.ie (E.Z.); 2 Fraunhofer Institute for Process Engineering and Packaging, Giggenhauser Str. 35, D-85354 Freising, Germany; 3 Department of Food Science, University of Copenhagen, 1958 Frederiksberg C., Denmark; ilp@food.ku.dk (I.L.P.); marcel@food.ku.dk (M.S.J.); 4 IFEU-Institut für Energie-und Umweltforschung Heidelberg GmbH, Im Weiher 10, 69121 Heidelberg, Germany; andreas.detzel@ifeu.de (A.D.); mirjam.busch@ifeu.de (M.B.); martina.krueger@ifeu.de (M.K.); 5 APC Microbiome Ireland, University College Cork, Ireland

**Keywords:** lupin, protein, functionality, FODMAPs, nutrition, digestibility, carbon footprint, life cycle assessment, sustainability

## Abstract

Similarly prepared protein isolates from blue lupin (*Lupinus angustifolius*) and white lupin (*L. albus*) were assessed in relation to their composition, functional properties, nutritional attributes and environmental impacts. Blue lupin protein isolate (BLPI) and white lupin protein isolate (WLPI) were found to be quite similar in composition, although differences in the electrophoretic protein profiles were apparent. Both lupin protein isolates (LPIs) had good protein solubility (76.9% for BLPI and 69.8% for WLPI at pH 7) and foaming properties. However, a remarkable difference in heat gelation performance was observed between BLPI and WLPI. WLPI had a minimum gelling concentration of 7% protein, whereas BLPI required 23% protein in order to form a gel. WLPI also resulted in stronger gels over a range of concentrations compared to BLPI. Nutritional properties of both LPIs were similar, with no significant differences in in vitro protein digestibility (IVPD), and both had very low trypsin inhibitor activity (TIA) and fermentable oligo-, di- and monosaccharides, and polyols (FODMAP) content. The amino acid profiles of both LPIs were also similar, with sulfur-containing amino acids (SAAs) being the limiting amino acid in each case. Environmental impacts revealed by the life cycle assessment (LCA) were almost identical for BLPI and WLPI, and in most categories the LPIs demonstrated considerably better performance per kg protein when compared to cow’s whole milk powder.

## 1. Introduction

World population is expected to grow by 2 billion by 2050, reaching 9.7 billion [1], and this will inevitably put pressure on food systems to meet the increased demand for dietary protein. Increasing animal production is an unsustainable option for meeting this demand, due to its high environmental impact [2,3]. Animal products (including meat, eggs and dairy) provide only 37% of dietary protein, yet take up around 83% of global farmland and generate up to 58% of emissions from food [4]. Since a proportion of plant protein is lost in conversion from feed to animal protein (85% in some cases) [5], there is a strong ethical case for increasing the use of plant protein crops directly for human consumption, rather than animal feed [6]. As a result, legumes such as lupin are increasingly being explored as alternatives to animal protein, and indeed to soy [2]. Lupins have traditionally been used as a source of food in the Mediterranean region as well as South America; the 4 main species consumed are *Lupinus angustifolius* (blue lupin or narrow leafed lupin), *L. albus* (white lupin), *L. luteus* (yellow lupin) and *L. mutabilis* (Andean lupin) [7,8]. Lupins are particularly attractive as a source of plant protein as they are higher in protein and lower in carbohydrate than other legumes, e.g., peas, chickpeas; the protein content of dehulled lupin seeds is in the region of 39%–55% of dry matter, comparable to soybeans [9]. Various techniques have been employed to produce lupin protein concentrates/isolates, including alkaline/neutral extraction followed by isoelectric precipitation (IEP) or ultrafiltration (UF), salt extraction/micellization, and air classification [10,11,12,13,14]. Lupin protein has been utilised in various food and beverage applications, such as plant-based milk or yogurt-type products, tempeh and mayonnaise [15,16,17,18,19,20,21]. There has also been much focus on lupin components due to their potential nutraceutical properties, including blood glucose and cholesterol modulating effects [22,23,24]. At the same time, the high fermentable oligo-, di- and monosaccharides, and polyols (FODMAP) content (galactooligosaccharides in particular) of lupins and other legumes [25] is a concern for individuals with irritable bowel syndrome (IBS) [26]. Another consideration for legumes is the presence of antinutritional compounds, which can negatively affect their nutritional value [27]. The aim of this paper was to compare the performance of protein isolates derived from blue and white lupin, with a holistic approach including techno-functionality, nutritional aspects, and environmental impacts. These properties are all important considerations if lupin is to become more widely incorporated in food systems as a sustainable, nutritious and functional plant protein source. 

## 2. Materials and Methods 

### 2.1. Chemicals and Raw Materials

All chemicals were purchased from Sigma-Aldrich (St. Louis, MO, USA) unless otherwise stated. *L. albus* cv. Butan seeds were used for the production of white lupin protein isolate (WLPI). In addition, blue lupin protein isolate (BLPI) from *L. angustifolius* cv. Boregine seeds (commercially produced by Prolupin) was used.

### 2.2. Preparation of Lupin Protein Isolate

Each lupin protein isolate (LPI) was produced as a single batch at industrial scale, based on previously reported methods [10,28]. For both BLPI and WLPI, lupin seeds were cleaned, de-hulled and sifted into hulls and protein-rich kernels as main fractions. Following this, the de-hulled seeds were flaked to give yellow flakes, which were subsequently de-oiled with supercritical CO_2_ to obtain white flakes as an input material for the extraction of protein. The resulting white flakes were enriched in protein (40%–50% protein) and had a low lipid content (<2%) compared to the seed material.

The first step of the aqueous protein extraction was an acid extraction of the lupin flakes in order to separate low molecular weight substances (e.g., oligosaccharides, alkaloids) in the acid extract from high molecular weight proteins and dietary fibres in the remaining pellet. This was carried out at pH 4.5 and 12 °C for a duration of 45 min, using HCl to adjust pH. In the case of BLPI, the acid extraction was repeated for a duration of 5 min to improve the sensory properties of the final product. The acid extraction was followed by a neutral extraction in which the high molecular weight proteins were extracted from the residual dietary fibre pellets at neutral pH. This was carried out at pH 7 and 30 °C for a duration of 30 min, using NaOH to adjust pH. The insoluble fibres were then separated from the protein extract containing soluble high molecular weight proteins by centrifugation with decanters. In the case of WLPI the neutral extraction was repeated for a duration of 5 min, in order to give a slight improvement in yield. Finally, the resulting protein extracts were heated to 35 °C and adjusted to pH 4.5 using HCl for precipitation of the proteins under isoelectric conditions, for a duration of 10 min. Subsequently the precipitated proteins were separated in a disc-type separator, neutralised with NaOH, pasteurised and spray-dried to obtain LPIs. The difference in repeated steps between BLPI and WLPI were not expected to have a major impact on LPI properties [29].

### 2.3. Compositional Analysis

Compositional analysis was carried out by Concept Life Sciences Ltd. using the following methods: protein content was analysed using the Dumas method using a nitrogen-to-protein conversion factor of 6.25 (protein content using N*5.7 is shown for comparison, as this conversion factor is also commonly used for lupin protein [12]); fat content was measured using low resolution proton nuclear magnetic resonance; saturated, mono-unsaturated, poly-unsaturated and trans fatty acids were quantified using Gas Chromatography–Flame Ionisation Detection (GC-FID) analysis; ash content was determined by oxidation at 550 °C to remove organic matter; moisture was determined by oven drying (105 °C) for a minimum of 16 h; sodium was determined using flame photometry after ashing at 550 °C; other minerals were analysed using inductively coupled plasma atomic emission spectroscopy or ion chromatography; dietary fibre content was analysed in accordance with the AOAC method 991.43 [30]. Amino acid composition was determined by Chelab S.r.l. using ion chromatography with post-column derivatization with ninhydrin, or HPLC-UV analysis in the case of tryptophan.

### 2.4. Protein Profile Analysis

An Agilent Bioanalyzer 2100 Lab-on-a-Chip capillary electrophoresis system was used to analyse the protein profile and estimate the molecular weights of the respective protein bands. Samples were prepared according to Amagliani et al. [31] with slight modifications: LPIs were dispersed in 2% SDS, 2 M thiourea and 6 M urea, to give a protein concentration of 2.5 mg/mL. Dispersions were shaken for 2 h at 22 °C, and centrifuged to remove insoluble material. Samples were analysed using an Agilent Protein 80 kit and Protein 230 kit according to the instructions within the ranges of 5–80 and 14–230 kDa, respectively. For reducing conditions, DTT was included in the sample buffer according to kit instructions.

### 2.5. Scanning Electron Microscopy 

Scanning electron microscopy (SEM) was carried out according to the method of Alonso-Miravalles et al. [32] using a JSM-5510 scanning electron microscope (JEOL Ltd., Tokyo, Japan).

### 2.6. Particle Size Distribution

Particle size distribution (PSD) of protein dispersions was measured using a static laser light diffraction unit (Mastersizer 3000, Malvern Instruments Ltd., Worcestershire, UK), covering a size range of 0.01–3000 μm. For the preparation of samples, LPIs were dispersed in ultrapure water in 50 mL centrifuge tubes at a concentration of 1% protein (*w/v*), pH adjusted to 7, and samples were shaken overnight at 4 °C. The particle refractive index was set at 1.45, the absorption used was 0.1 and the dispersant refractive index was 1.33. Protein dispersions, equilibrated to 22 °C, were introduced into the dispersing unit using ultrapure water as dispersant until a laser obscuration of 12% was achieved. Results were presented as volume-weighted mean particle diameter (D_4,3_), surface-area weighted mean particle diameter (D_3,2_) and volume percentiles (Dv(10), Dv(50), Dv(90)). 

### 2.7. Surface Hydrophobicity 

Surface hydrophobicity (S_0_) was measured based on the method of Hayakawa and Nakai [33] using 1-anilino-8-naphthalenesulfonate (ANS) with slight modifications as described by Karaca et al. [34]. Protein dispersions were serially diluted with 10 mM phosphate buffer (pH 7) in the range of 0.0006–0.015% (*w/v*). ANS (10 µL; 8.0 mM in 0.1 M phosphate buffer, pH 7) was mixed with 2 mL diluted sample and left in darkness for 15 min. Fluorescence was measured (λ_excitation_ 390 nm, λ_emission_ 470 nm) and corrected by a blank measured without ANS. The results are presented as the slopes (*r*^2^ ≥ 0.98) of the absorbance versus protein concentration.

### 2.8. Protein solubility

Protein solubility, as influenced by pH, was evaluated using the Kjeldahl method. First, protein content (N*6.25) of LPIs was measured, dispersions of 1% (*w/v*) protein were prepared, and pH was adjusted from 3.0 to 8.0 in 0.5 pH unit intervals using HCl or NaOH. Dispersions were hydrated at 4 °C overnight. Samples were then adjusted to 22 °C while shaking and pH was re-adjusted if necessary. Samples were centrifuged at maximum speed (4893× *g*) for 30 min. The protein content of the supernatants was then measured and protein solubility was expressed as % of original protein content in the dispersion remaining in the supernatant.

### 2.9. Zeta Potential 

The zeta potential of protein dispersions over the same pH range as for protein solubility analysis were determined using a Zetasizer nano-Z (Malvern Instruments Ltd.; UK). Samples (0.1% *w/v*) were prepared in ultrapure water and pH was adjusted using HCl or NaOH. Samples were shaken overnight at 4 °C, adjusted to 22 °C and pH was readjusted if necessary. Samples were then centrifuged at 2000× *g* for 10 min to remove any insoluble material. The measurement was performed using an automatic voltage selection and zeta potential was calculated using the Smoluchowski model. A refractive index and absorption of 1.45 and 0.001 were used, respectively.

### 2.10. Foaming Properties

Foaming properties were assessed according to the method of Alonso-Miravalles et al. [32]. Dispersions (20 mL in 50 mL centrifuge tubes) with a protein concentration ranging from 0.1% to 3.3% (*w/v*) in 0.1 M phosphate buffer pH 7 were frothed using an Ultra-Turrax equipped with a S10N-10G dispersing element (Ika-Labortechnik, Janke and Kunkel GmbH, Staufen, Germany) at maximum speed for 30 s. The height of the sample (liquid and foam phase) was measured immediately, and after 60 min. Foaming capacity was taken as % sample expansion at 0 min, while foam stability was taken as sample expansion at 60 min as a percentage of sample expansion at 0 min Sample expansion was calculated using the following equation:

Sample expansion (%) = ((Sample height after foaming − initial sample height)/Initial sample height) × 100(1)

### 2.11. Minimum Gelling Concentration

Minimum gelling concentration of each protein was determined using protein dispersions in 10 mM phosphate buffer (pH 7) over a range of concentrations. Dispersions (5 mL) were prepared in 15 mL centrifuge tubes and hydrated overnight at 4 °C. Tubes were heated at 90 °C in a water bath for 30 min, cooled rapidly under running water, and maintained overnight at 4 °C. Tubes were then inverted and the minimum protein concentration at which the dispersion did not flow was taken as minimum gelling concentration.

### 2.12. Rheological Analysis of Heat Gelation Properties

Rheological tests were carried out using a controlled stress rheometer (MCR301, Anton Paar GmbH, Graz, Austria) equipped with a concentric cylinder measuring system (C-CC27-T200/SS, Anton Paar GmbH, Austria). Protein dispersions (10, 15, 20 and 25% *w/v*) were hydrated overnight at 4 °C, adjusted to 22 °C, sheared for 10 s at speed 3 with an Ultra-Turrax T10 equipped with a S10N-10G dispersing element (Ika-Labortechnik, Janke and Kunkel GmbH, Staufen, Germany) to ensure there were no lumps, and pH was then adjusted to 7.0. Small deformation oscillatory rheology was used to monitor heat gelation with strain and frequency of 0.1% and 1 Hz, respectively. The temperature profile used was as follows: temperature was increased from 20 to 90 °C at 2 °C/min, held at 90 °C for 30 min, cooled to 20 °C at 2 °C/min and held at 20 °C for 30 min. This was followed by a logarithmic frequency sweep from 0.01 to 10 Hz, maintaining strain at 1%. Following this, the large deformation properties of the gels were examined by applying rotational shear at a shear rate of 0.005 s^−1^, and stress/strain curves were generated [35], from which the gel fracture properties could be assessed.

### 2.13. In Vitro Protein Digestibility

Simulated gastro-intestinal protein digestion was performed using a multistage, static in vitro protein digestibility (IVPD) method, according to previous descriptions [36,37]. In short, LPI samples were initially weighed to contain 50 ± 0.1 mg protein on dry matter basis. Enzymatic hydrolysis consisted of pepsin digestion (1 h, 37 °C) followed by a sequential short-term pancreatin digestion (1 + 1 h, 37 °C). Enzyme to substrate (E:S) ratios applied during the stages of pepsin and pancreatin digestion were kept constant at 1:50 and 1:10 *w/w*, respectively. IVPD % was determined using a trinitrobenzenesulfonic acid (TNBS)-based quantification method, applying an alanine standard solution representing 100% protein digestibility at each stage of digestion [36].

### 2.14. Trypsin Inhibitor Activity (TIA) Assay

Trypsin inhibitors were extracted from the LPIs by weighing of the samples (350 mg) and dilution in sodium acetate buffer (2.5 mL, 0.1 M, pH 4.9), followed by Ultra-Turrax homogenization for 2 min. The samples were centrifuged at 3000× *g* for 5 min (EBA 12 Centrifuge; Hettich Zentrifugen, Tuttlingen, Germany) and the supernatants were transferred into new test tubes. The residual pellet was resolubilised, homogenised, and centrifuged to repeat the procedure under identical conditions. The supernatants were pooled, maintained at 4 °C overnight, and centrifuged again at 3000× *g* for 5 min before analysis of TIA. TIA levels of the LPI were measured, with a few modifications, according to a previously described method [36]. Briefly, the TIA levels were determined against a solution of purified trypsin enzyme with a stock concentration of 0.1 mg/mL. The substrate solution used in this assay was 0.22 mg/mL *N*-α-benzoyl-l-arginine-4-nitroanilide (l-BAPA). The product (4-nitroaniline), with a molar extinction coefficient of 8800 M^−1^ × cm^−1^ was used for spectrophotometric quantification at 410 nm. In this assay, 1 trypsin inhibitor unit (1 TIU) is defined as the amount of inhibitor required to reduce the enzyme activity by 1 trypsin activity unit (TU). TU is defined as the amount of enzyme that catalyses hydrolysis of 1 μmol of l-BAPA into the product (4-nitroaniline) in 1 min at pH 8.2 at 37 °C. TIA levels of the samples were calculated based on sample or protein mass on dry weight basis, and expressed as TIU/mg sample DM or TIU/mg protein DM.

### 2.15. Quantification of Fermentable Oligo-, Di- and Monosaccharides, and Polyols

The quantification of mono-, di-, galactooligosaccharides, fructans, and polyols was conducted using high performance anion-exchange chromatography coupled with pulsed amperometric detection (HPAEC-PAD), performed on a DionexTM ICS-5000+ system (Sunnyvale, CA, USA), as described by Ispiryan et al. [38]. All carbohydrates, except for the fructans, were quantified using authentic reference standards, as specified in the previous study [38]. The total fructan content was determined after enzymatic hydrolysis with two enzyme mixtures A and B, where only B contained fructan degrading inulinases. The calculation was based on the quantification of the monomers glucose and fructose released from the fructan molecules [38]. The significance of the fructose released from sucrose and the fructose released from the hydrolysis with the enzyme mixture B has been determined. If no significant difference was determined and all levels below 0.1 g/100 g are referred to as not detected (n.d.) in further discussions [39]. 

All extractions were carried out in duplicate according to the method described by Ispiryan et al. [38]. The results of the ingredients are presented in g analyte per 100 g sample on a dry weight basis (g/100 g DM).

### 2.16. Life Cycle Assessment

Environmental performance of LPIs was examined by means of life cycle assessment (LCA) using Umberto 5.5 software. LCA was carried out as an attributional cradle-to-gate LCA and includes the individual processes associated with LPIs as shown in Figure 10. Impact assessment methods are based on Umweltbundesamt Berlin [40]. Microsoft Excel 2010 and Microsoft PowerPoint 2010 were used in the production of graphics.

### 2.17. Statistical Data Analysis

Unless otherwise stated, all analyses were carried out in triplicate, with the exception of compositional analyses, which were performed following a validated method and therefore analysed just once and reported without standard deviation. In the case of the amino acid analysis, validated uncertainty values are included. Results were subjected to two-tailed, unpaired Student’s t-test to determine statistically significant differences (*p* < 0.05) between mean values for the different samples. The statistical programs used were IBM SPSS version 26 (Armonk, NY, USA) or GraphPad Prism version 8.3.0. (San Diego, CA, USA) The results are presented as mean ± standard deviation where applicable. 

## 3. Results and Discussion

### 3.1. Compositional Analysis

The results of the macronutrient and micronutrient compositional analysis for BLPI and WLPI are shown in Table 1. The composition of both ingredients were broadly similar, however, there were some small differences, including a slightly higher protein content for WLPI. Both LPIs had protein content of >90%, which is comparable to that typical of dairy protein isolates [41]. Both LPIs had a low fat content (close to 1%) which was expected, as defatted flakes were used as starting material for the protein isolation. Dietary fibre was not detected in either LPI, indicating it was effectively removed during the isolation process. The mineral profile was quite similar for both LPIs, but with some differences, such as higher iron level for BLPI and higher manganese level for WLPI. Both LPIs were relatively high in sodium; this may have resulted from the addition of NaOH is used for neutralisation prior to drying [32]. The high sodium content may limit the amount of suitable applications for such ingredients. 

### 3.2. Structural and Surface Properties

#### 3.2.1. Protein Profile

**Figure 1:**
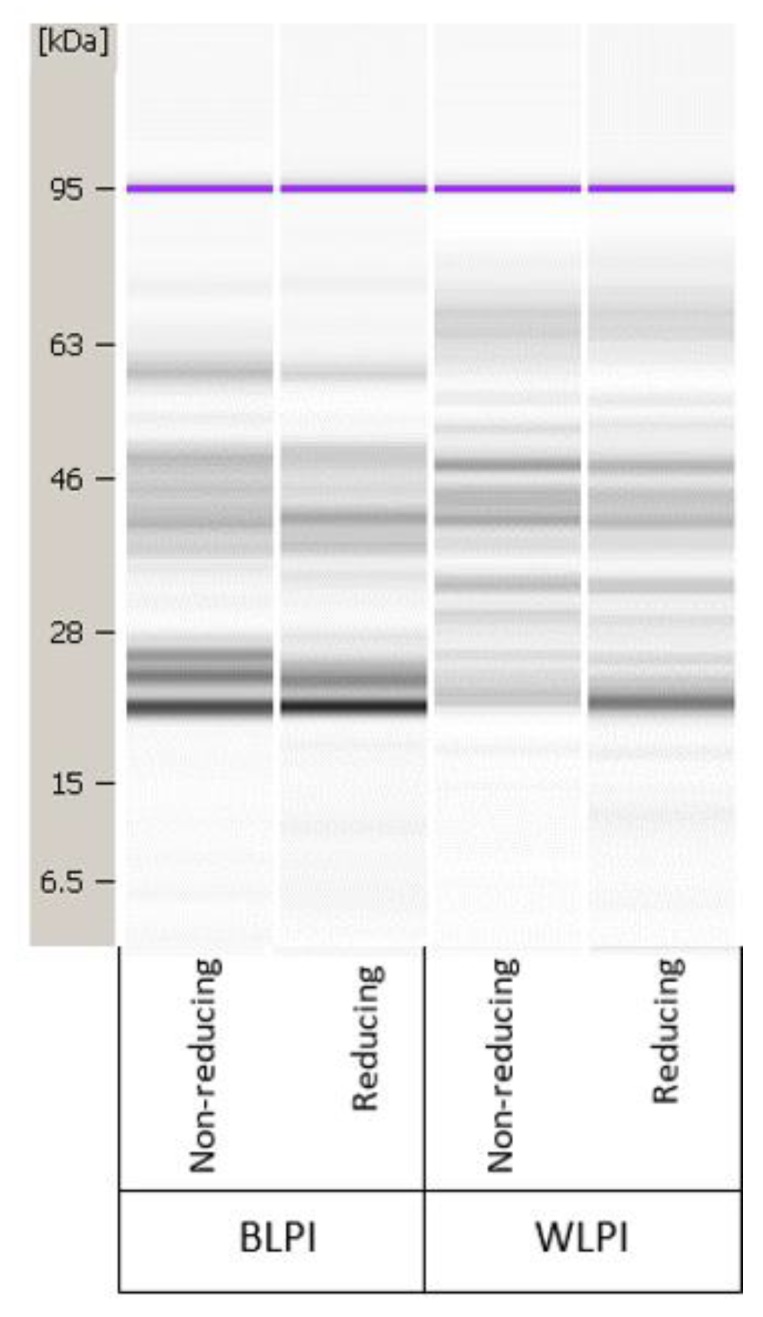
Representative protein profile for blue lupin protein isolate (BLPI) and white lupin protein isolate (WLPI) under non-reducing and reducing conditions, in the range of 5–80 kDa.

The protein profiles of BLPI and WLPI under reducing and non-reducing conditions from the Bioanalyzer analysis are shown in Figure 1. Results in the 5–80 kDa range only are shown, since no additional bands were present in the higher molecular weight range analysed. The proteins found in lupins share structural similarities with other legume proteins; they are mainly storage proteins, with the majority being globulins [5,42]. The 4 main fractions in lupin seed proteins are: α-, β-, δ- and γ-conglutins. Approx. 80% of the protein is comprised of α-conglutin (11s legumin-like globulins) and β-conglutin (7s vicilin-like globulins) [43]. Gamma-conglutin, which mainly remains in the acid soluble fraction during the IEP process, is of interest for nutraceutical applications, but also has good foamability, while α- and β-conglutin are of most interest from a techno-functional perspective [44]. Alpha- and β-conglutin are present in native form as hexamers of 330–430 kDa and trimers of 143–260 kDa, respectively, the monomer/subunits of which are most likely represented in Figure 1. Each α-conglutin monomer is comprised of an acidic (45–52 kDa) and basic (20–22 kDa) subunit, linked by a disulfide bridge. Beta-conglutins are comprised of low, intermediate and high molecular weight monomers within the range of 17–64 kDa [43]. The bands shown for BLPI and WLPI fall into these ranges; however, the two profiles are noticeably different, with the WLPI bands more complex and diffuse across the range. This may be expected since these isolates are derived from different species, and much heterogeneity between conglutin subunits has been observed, even within the same cultivar [45]. Some differences can be seen between non-reducing and reducing conditions for both BLPI and WLPI, mainly more intense bands around 22 kDa for BLPI and WLPI, and around 40 kDa for BLPI, suggesting some dissociation of disulfide bonds and dissociation of α-conglutin into its subunits under reducing conditions, which was more apparent in WLPI than in BLPI.

#### 3.2.2. Scanning Electron Microscopy

**Figure 2:**
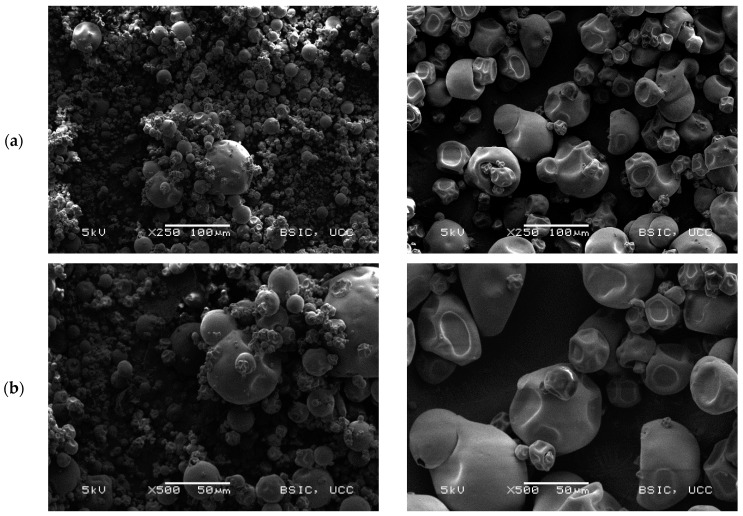

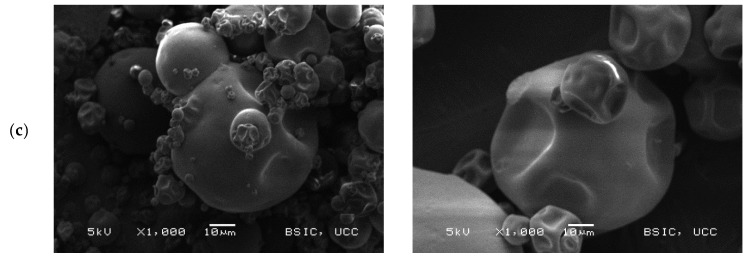
Representative scanning electron microscopy micrographs of BLPI (left column) and WLPI (right column). Magnifications shown are (**a**): 250×, (**b**): 500× and (**c**): 1000×.

Scanning electron microscopy (SEM) images (Figure 2) show the microstructural characteristics of the BLPI and WLPI powders over a range of magnifications. The particles observed were roughly spherical, with a shrunken but smooth appearance typical of spray dried high protein powders [32,46,47]. BLPI consisted of a greater proportion of smaller particles which are clustered together, with a smaller proportion of larger particles, whereas the greater proportion of WLPI was observed to be comparatively large particles, which were less closely arranged. 

#### 3.2.3. Particle Size Distribution of Dispersions 

**Figure 3:**
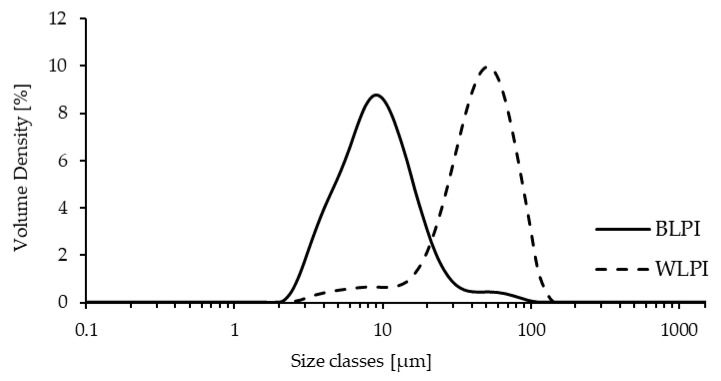
Volume weighted particle size distribution for BLPI and WLPI after overnight hydration at 4 °C.

The volume weighted particle size distributions for BLPI and WLPI dispersions after overnight hydration are shown in Figure 3. The distribution for both LPIs was monomodal. A similar size range of approximately 2–130 μm was apparent for both BLPI and WLPI. However, the volume distribution was dominated by smaller particles for BLPI, and larger particles for WLPI, which also corresponds the SEM micrographs. The values for D_4,3_, D_3,2_, Dv (10), Dv (50) and Dv (90) are shown in Table 2, where in each case the value was significantly larger for WLPI. These results indicate that large, poorly dispersible particles remained present after overnight hydration, in an approximately similar size range as has been observed with spray dried lentil protein isolates [32] and milk protein concentrates [48]. It is worth noting that in applications such as non-dairy beverages or ice creams, high pressure homogenisation is likely to be applied, which may reduce particle size and improve dispersability [49,50].

#### 3.2.4. Surface Hydrophobicity

Surface hydrophobicity (S_0_), which is dependent on the extent of exposed hydrophobic regions on the proteins, is an important property, which can be correlated with functional properties including emulsification and foaming [51,52]. S_0_ was significantly higher for BLPI (2185 ± 67.0) compared to WLPI (842 ± 274 (Table 2). Lampart-Szczapa et al. [53] measured the S_0_ of untreated soluble protein from lupin seeds using a similar method. They found S_0_ values of 822, 959 and 813 for *L. luteus*, *L. albus* and *L. angustifolius* varieties respectively, with hulls excluded. These values are similar to that found for WLPI here, whereas S_0_ for BLPI was considerably higher. This difference may have resulted from inherent structural differences between proteins from the two species of lupin. Alternatively, greater surface area due the prevalence of smaller particles in BLPI compared to WLPI may have been responsible for the higher S_0_ value. Additionally, if BLPI experienced a greater degree of denaturation during processing than WLPI, this could also have contributed to higher S_0_. 

### 3.3. Techno-Functional Properties 

#### 3.3.1. Protein Solubility and Zeta Potential

**Figure 4:**
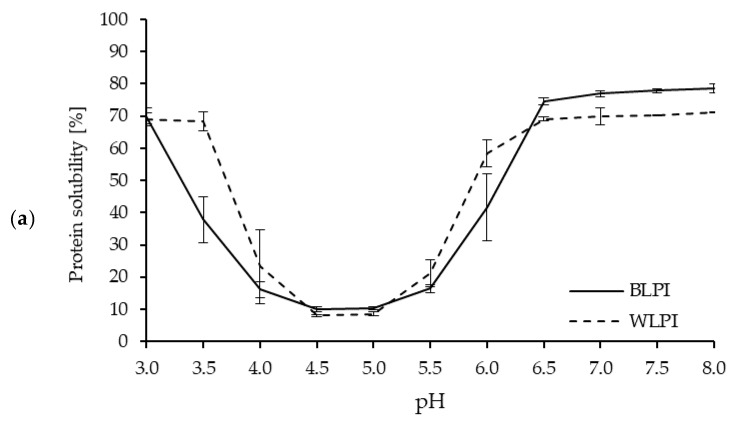

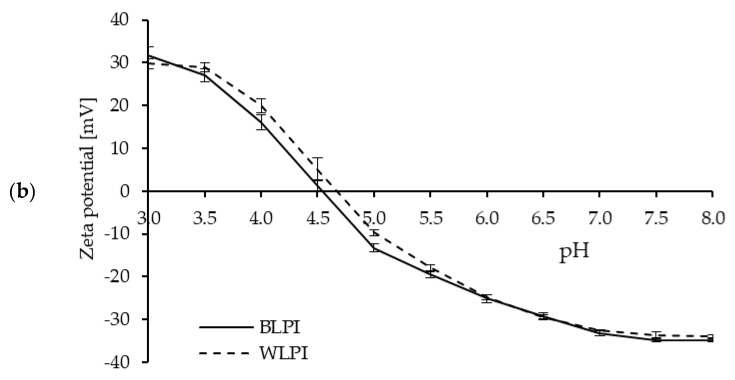
Protein solubility (**a**) and zeta potential (**b**) as a function of pH for BLPI and WLPI (error bars show standard deviation). Significant differences (*p* < 0.05) were found between BLPI and WLPI for protein solubility (**a**) at pH 3.5, 4.5, 5, 6.5, 7, 7.5 and 8, and for zeta potential (**b**) at pH 5, 7.5 and 8.

Protein solubility is an important property for many food and beverage applications, and is often a prerequisite for various functional properties, such as gelation, emulsification, and foam formation [5,54]. Protein solubility, as influenced by pH, for both BLPI and WLPI followed a similar pattern to previous results for high protein ingredients based on lupin [55,56] and other legumes [57], with solubility decreasing as pH approached the isoelectric point (pI), around pH 4.5–5, where minimum solubility (8%–10%) was observed (Figure 4). This corresponds with the zeta potential results, where a zeta-potential of 0 mV was observed around pH 4.5 for BLPI and WLPI. One notable difference was the higher solubility for WLPI at pH 3.5, this may be of interest for some acidic beverage or food applications. The effect of pH on solubility was minimal for both LPIs between pH 6.5–8. Protein solubility at pH 7 was 76.9% for BLPI and 69.8% for WLPI; significantly higher for BLPI, although not a major difference. Similar values for solubility at pH 7 have been reported elsewhere for LPIs produced by IEP [58,59,60].

#### 3.3.2. Foaming Properties

**Figure 5:**
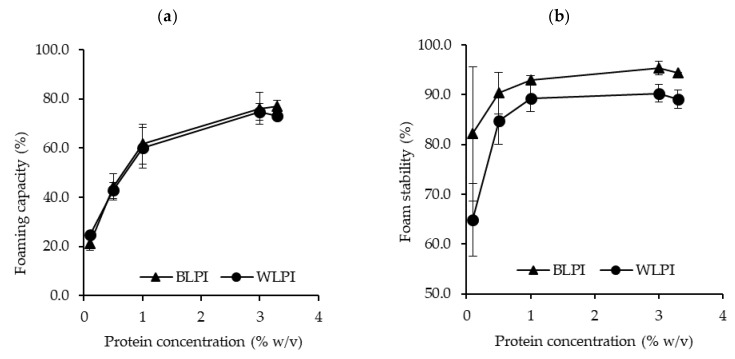
Foaming capacity (**a**) and foam stability (**b**) for BLPI and WLPI in the range of 0.1%–3.3% protein, at pH 7 and 22 °C (error bars show standard deviation). No significant differences (*p* < 0.05) were found between BLPI and WLPI for foaming capacity (**a**). Significant differences (*p* < 0.05) were found between BLPI and WLPI for foam stability (**b**) at 3 and 3.3% protein.

The foaming capacity and foam stability for BLPI and WLPI over a range of concentrations are shown in Figure 5. BLPI and WLPI showed very similar patterns for foaming capacity, with higher foaming capacity as the concentration increased. Both LPIs reached a foaming capacity of >70% at 3 and 3.3% protein. Foam stability was also similar for both LPIs after 1 h, close to 90% at protein concentrations greater than 1%, and slightly but significantly higher values were found for BLPI at 3 and 3.3% protein. The relatively higher surface hydrophobicity of BLPI does not seem to be reflected in the foaming properties as both LPIs displayed similar results. Literature values for foaming properties of LPIs vary, e.g., Chew et al. [55] reported very low foaming capacity, Piornos et al. [61], reported foaming capacity of 89.29% and foam stability of 43.91% at a concentration of 2% protein while D’Agostina et al. [10] reported foaming capacity well over 1000% for 5% dispersions, albeit different equipment and methodology was used between studies. From the results here, it appears that BLPI and WLPI have relatively good foaming properties at neutral pH, and may be suitable for applications where foam formation is desired e.g., non-dairy desserts, and some bakery applications.

#### 3.3.3. Gelation Properties

**Figure 6:**
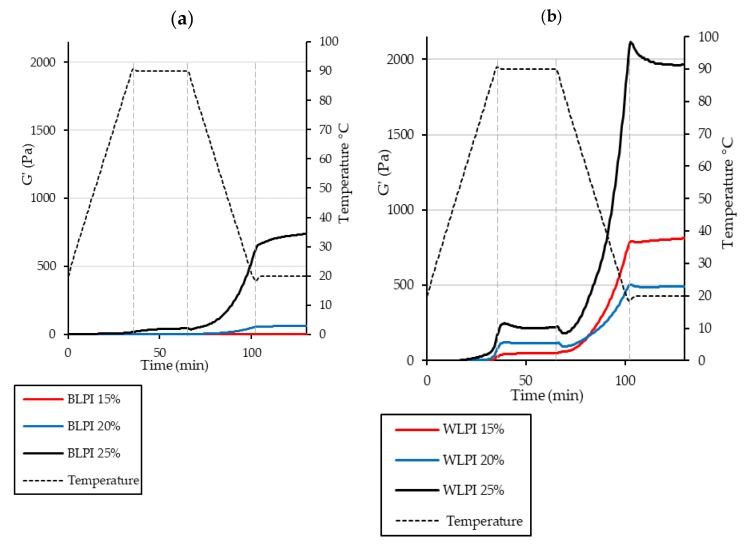
Rheological temperature sweeps for BLPI (**a**) and WLPI (**b**) at protein concentrations in the range of 15%–25%, showing storage modulus (G’) and temperature. Curves show the average values of triplicate analysis.

Gelling ability of proteins is an important requirement for many food products. Traditionally egg, dairy or soy proteins have commonly been used for such applications; however, interest is growing in the use of alternative legume proteins for this purpose [62,63]. Thermal gelation of globular proteins involves molecular unfolding on heating, aggregation of proteins resulting from newly exposed groups to minimise the energy of the system, and formation of a continuous protein network providing a more elastic structure [62,64,65]. The minimum protein concentration for gelation was found to be 23% for BLPI and 7% for WLPI. At these concentrations, the dispersions did not flow on inversion, although the gels still appeared weak. This is in agreement with the results of Bader et al. [50] where protein isolate from white lupin formed a gel at 15% protein after heating at 95 °C for 1 h, whereas protein isolate from blue lupin did not. This difference in concentration dependence was also evident in the rheological temperature sweeps: storage modulus (G’) describes the elastic response of the material during oscillation, whereas loss modulus (G”) describes the viscous response. Gel development during heating and cooling is shown in Figure 6, with G’ displayed only, for clarity of presentation. Results for BLPI and WLPI 10% are not shown, as no gelation was evident for BLPI at this concentration. Values were considerably lower for BLPI at all concentrations compared to WLPI, demonstrating poorer gelling performance as a function of concentration. However, a similar pattern was observed for both BLPI and WLPI: an initial increase in G’ during the heating ramp, little or no strengthening during the holding period at 90 °C, and the strengthening mainly occurring during the cooling ramp. Similar behaviour on heating and cooling has been observed with LPI [66] and other plant proteins including soy, pea, cowpea and quinoa [63,67,68,69,70]. 

**Figure 7:**
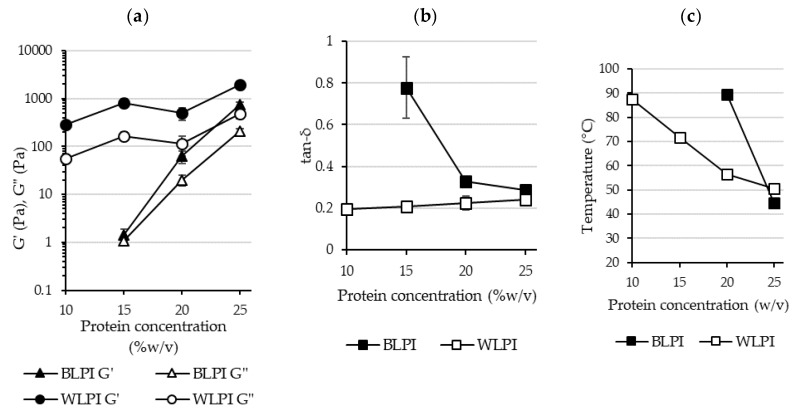
Final values are shown for storage modulus (G’) and loss modulus (G”) (**a**) and tan-δ (**b**) (error bars show standard deviation). Crossover temperatures taken from average G’ and G” curves are shown in (**c**) (not shown for BLPI 15% as crossover did not occur until the cooling phase).

The final G’, G” and tan-δ values after cooling and holding at 20 °C are shown in Figure 7. The strongest gel was formed with WLPI at 25% protein. Interestingly, WLPI at 20% did not result in a stronger gel than WLPI at 15% protein. For BLPI, 25% protein was required to reach G’ values similar to WLPI at 15% protein. Tan-δ was close to 0.2 for WLPI at all concentrations measured (Figure 7), increasing only slightly with concentration, indicative of the formation of elastic structures. Values were higher for BLPI, especially at 15%, where tan-δ was closer to 1, showing only limited elasticity. In the study of Berghout et al. [71], LPI gel at 15% protein had a similar G’ value to that of BLPI at 15% protein in this study. Interestingly, the G’ value of LPI gel at 24% protein in that study was higher than G’ for WLPI at 25% protein in this study, although different equipment and conditions were used. Crossover temperature, where G’ becomes greater than G”, can be taken as the temperature at which onset of gelation occurs, although this is likely dependent on heating rate [68]. Generally, it can be seen that this occurred at lower temperatures as concentration increased (Figure 7). In the case of 12% chickpea protein, crossover temperatures of ~65 °C have been observed, depending on the variety used [72]. However, when denaturation temperatures of LPI produced by IEP were assessed by Muranyi et al. [58], the first endothermic transition did not begin until well above 70 °C. This suggests that the lower crossover temperatures observed at higher protein concentration for BLPI and WLPI were not related to denaturation. Perhaps increased interaction of protein particles due to swelling upon heating could provide an explanation for this behaviour [71]. 

**Figure 8:**
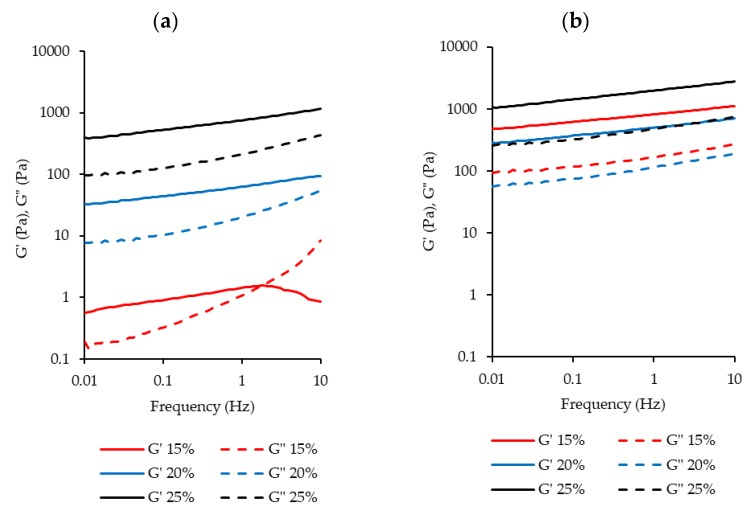
Rheological frequency sweeps from 0.1–10 Hz for BLPI (**a**) and WLPI (**b**) in the range of 15%–25% protein. Curves show the average values of triplicate analysis.

The mechanical spectra from frequency sweeps (Figure 8) also show the difference in gelation properties between BLPI and WLPI. For WLPI, G’ was greater than G” across the range of frequencies tested, and both G’ and G” were relatively independent of frequency, indicating formation of a cross-linked gel, while for BLPI the moduli were more frequency dependent, indicating weaker or limited gel formation [62,73]. Additionally, For BLPI at 15% protein, G” crossed above G’ at higher frequency.

**Figure 9:**
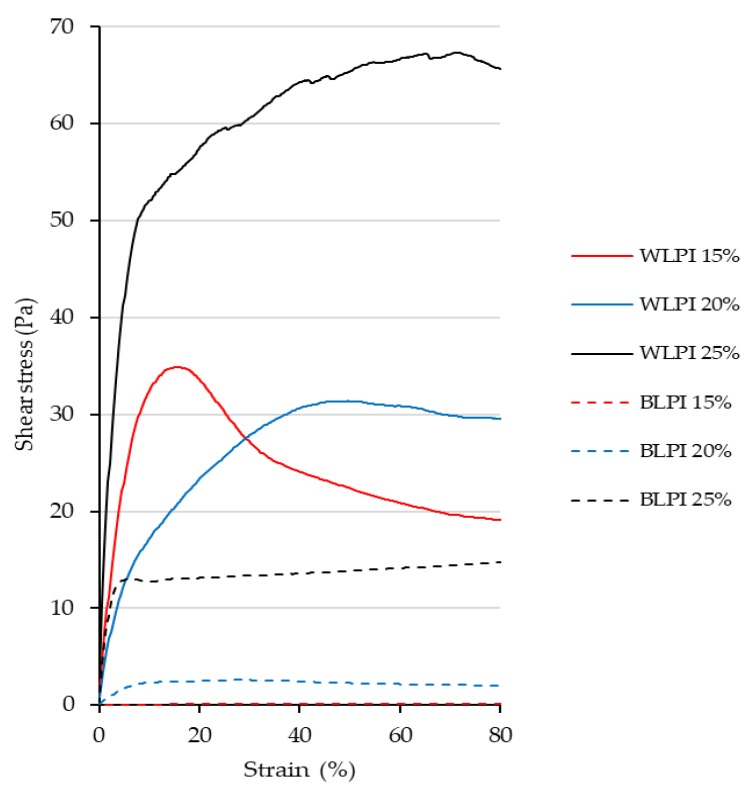
Rotational stress/strain curves for BLPI and WLPI gels at a shear rate of 0.005 s^−1^. Curves show the average values of triplicate analysis.

The large deformation properties for BLPI and WLPI gels are shown in the form of rotational shear stress vs strain curves in Figure 9. Large deformation properties of foods are important as they influence how products are experienced by the consumer, e.g., during handling and consumption [74]. It can be seen that BLPI gels were more easily deformable at all concentrations, and WLPI gel at 25% protein showed the highest stress and strain values, corresponding to the small deformation results. The type of curve varied depending on the sample and concentration. WLPI at 15% protein showed a reasonably clear fracture point. A clear fracture point was not apparent for WLPI at 20 and 25% protein, where stress decreased more gradually with applied strain. BLPI at 25% protein had an initial slope comparable to WLPI at 15% protein; however, the gel was broken at a considerably lower stress. It can be seen that at higher concentrations, as the gels were sheared/broken, they retained a viscous, ‘paste like’ consistency. This was also evident on examination of the gels; though self-supporting gels could be produced, particularly with WLPI, they were soft and easily deformable. This is in line with previous studies on gelation of lupin proteins, typically lupin protein gels are weaker and softer than soy protein gels and pea protein gels, and require higher protein concentrations to gel [62,66,71]. Minimum gelling concentrations of 11.5, 14 [66] and 16% [62] have been reported, suggesting there is a relatively wide range of gelling ability for LPIs. Previous studies have shown that the poorer gelling ability of lupin protein in comparison to soy protein may be due to the heat-stable nature of lupin proteins and their relative inability to form new disulfide bonds [62,71]. Kiosseoglou et al. [66] showed that reheating of lupin gels effectively reversed the strengthening effect of cooling, showing that the network formation and strengthening was due entirely to physical interactions, although the maximum temperature applied was 80 °C, however, in other tests they also demonstrated more covalent interactions in gels formed at 90 °C. The difference in gelling ability between BLPI and WLPI is remarkable, and may be due to an inherent higher heat stability for BLPI, resulting from possible structural differences as apparent in the protein profile. Additionally, there were some slight differences in the mineral profile, including a higher sodium content in BLPI, which could result in a decrease of electrostatic interactions due to shielding effects; however, this effect is unlikely to account for such a large difference [66].

### 3.4. Nutritional Properties

#### 3.4.1. Amino Acid Profile

The amino acid (AA) profiles for BLPI and WLPI (Table 3) were very similar. Overall, these profiles were consistent with previously reported data for lupin proteins [56,75]. The indispensable AA contents for BLPI and WLPI are also shown in Table 3 as a percentage of the WHO (2007) adult requirements [76]. Based on these requirements, sulfur-containing amino acids (SAAs) (methionine and cysteine) were limiting for both BLPI and WLPI, providing 66 and 62% of the requirement, respectively. Both BLPI and WLPI were slightly deficient in lysine and valine, while WLPI was also slightly deficient in tryptophan. Furthermore, the combined value for phenylalanine and tyrosine was higher for WLPI compared to BLPI. Lupin proteins, as well as most other legume proteins, are generally relatively low in SAAs [55,56,77]. However, literature values vary for SAA content, particularly with blue lupin; for flours, values of 1.52–4.8 g/100 g protein have been reported, and for protein isolates values of 1.61–4.2 g/100 g protein have been found [55,78,79]. In the case of white lupin, values of 2.58 and 2.64 g/100 g protein have been reported for flour [80,81] and 1.68 g/100 g protein for protein isolate [56]. The proportion of SAAs has been found to increase [42,55,78] and also to decrease [79] during isolation of lupin proteins, indicating that at least some of the variation observed is due to differences in processing conditions. Additionally, Sujak et al. [75] compared AA profiles of whole lupin beans of various cultivars, and on average white lupins were found to be slightly higher in SAAs than blue lupins. The implications of AA profile may vary depending on diet; it is of particular importance where diets are poor or protein sources are limited. The AA score of BLPI and WLPI may be improved if blended with other plant proteins such as cereal proteins rich in SAAs [6].

#### 3.4.2. In Vitro Protein Digestibility and Trypsin Inhibitor Activity

In vitro protein digestibility (IVPD) and trypsin inhibitor activity (TIA) of LPIs are presented in Table 4. The pepsin digestibility values ranged from 3.2%–3.7%, whereas the overall short-term protein digestibility values ranged from 35.7%–36.8%. BLPI and WLPI showed a statistically equivalent pepsin digestibility (*p* = 0.34) and short-term digestibility (*p* = 0.46), indicating a similar susceptibility of the lupin proteins towards transient enzymatic hydrolysis, irrespective of the lupin species. The average peptide chain lengths (APCL = 100%/IVPD %) of BLPI following pepsin and short-term digestion were 31.0 and 2.7 AAs, respectively. The corresponding APCL values of digested WLPI were 27.2 and 2.8 AAs, respectively. TIA levels were significantly lower in WLPI compared to BLPI (*p* < 0.05), both based on sample and protein mass. However, both LPIs showed extremely low TIA levels compared to other plant-based protein ingredients. Based on these results, it appears that blue and white lupin perform equally well as protein sources, in relation to digestibility.

#### 3.4.3. FODMAP Analysis

Lupin seeds, and pulses in general, are known to contain high amounts of FODMAPs, of which galactooligosaccharides (GOS) are the most abundant non-digestible, fermentable sugars, contributing up to 10% of the total dry matter [25]. The threshold GOS concentration to trigger gastrointestinal symptoms in IBS patients was estimated to be 0.3 g per food serving [26], thus, the consumption of pulses is usually associated with gastrointestinal discomfort. Andersen et al. [82] determined 9.1 ± 2.6 g/100 g DM GOS in different varieties of lupin seeds. Similar levels were reported by Ispiryan et al. [39] in a commercial lupin protein concentrate (10.74 g/100 g DM), while in contrast, only traces of the lupin GOS were found in BLPI as well as WLPI (Table 5) [39].

Joehnke et al. [83] compared the FODMAP contents in lentil protein isolates acquired by UF or IEP. Whereas UF effectively removed compounds with molecular weight less than 10 kDa, including GOS, from the protein isolate, the IEP protein isolate still contained ~50% of the original GOS found in the starting material. During the IEP-processing, removal of GOS is most likely to occur with removal of the supernatant after acid precipitation. However, residual liquid in the precipitated protein material presumably contains GOS. In contrast to the lentil protein IEP-preparation process [32], the process for BLPI and WLPI includes 2 main acidic steps where GOS are likely to be solubilised and removed. Finally, both ingredients are suitable for any low FODMAP product formulations, thereby widening the potential reach of their nutritional benefits.

### 3.5. Life Cycle Assessment

**Figure 10:**
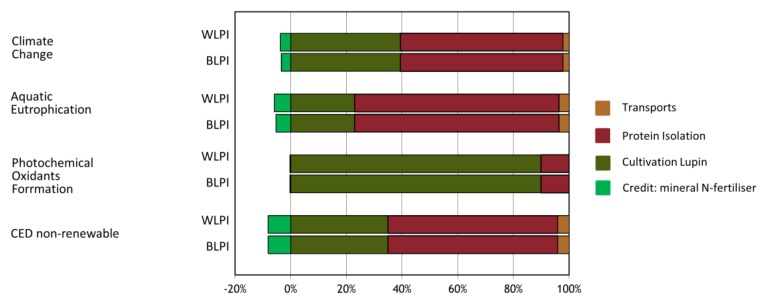
Contributions of main life cycle steps to environmental impact profiles of BLPI and WLPI. CED = Cumulative primary energy demand.

Environmental performance of BLPI and WLPI was examined by means of LCA (Table 6). Both Climate Change results for BLPI and WLPI and further indicator results were relatively close. Small differences indicate that potential environmental impacts are slightly higher for BLPI than for WLPI if compared on a protein mass basis. This observation is for the most part related to the small difference in protein content of the obtained LPIs. The difference in repeated steps between BLPI and WLPI corresponded to a very small difference in water use, with a negligible effect overall on the LCA results. The most relevant life cycle steps within the environmental profile of BLPI and WLPI are the cultivation phase (agriculture) as well as the processing from seeds up to LPI powder, depending on the indicator. The corresponding range in contributions of those life cycle steps to the overall indicator result is illustrated in Figure 10.

**Figure 11:**
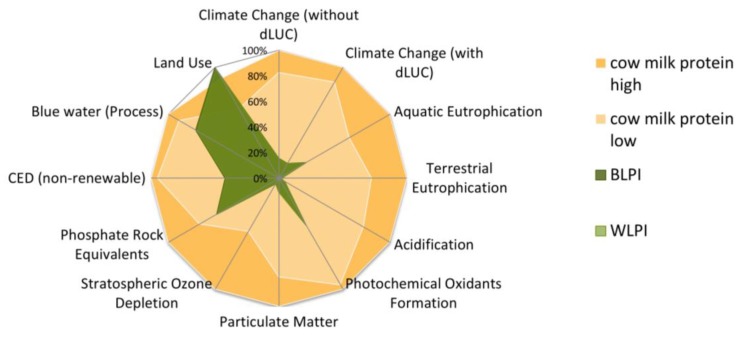
Comparison of environmental impact profiles of blue (BLPI) and white (WLPI) protein isolate powder versus cow’s whole milk powder ranges, per kg protein. Highest result is set to 100%. dLUC = direct land use change. LPI results are very close together, thus they have a complete graphical overlay in the chart.

The environmental impact profiles of BLPI and WLPI were also compared with traditional cow’s milk protein ranges (in the form of whole milk powder). The amount of feed per kg milk and share of concentrate versus silage feed are the basis for the two examined ranges. BLPI and WLPI are associated with lower potential environmental impacts than their cow’s milk-based counterpart for all indicators except the land use result. The latter finding is related to lower yields in lupin cultivation in a comparison with typical (cow) feed crops. Another point regarding this aspect is the economic value of sidestreams, which may increase in the future as commercial production begins to evolve further. This will also reduce the land use requirements associated with BLPI and WLPI. A comparative illustration of environmental performance (based on all examined indicators) of BLPI and WLPI versus cow’s milk protein is found in Figure 11.

## 4. Conclusions

Structural, functional and nutritional characteristics of BLPI and WLPI were examined, along with LCA, in order to compare blue and white lupin in terms of suitability for production of functional and sustainable protein isolates. The composition of both LPIs was similar, but differences were observed in their electrophoretic protein profiles. Protein solubility followed a similar pattern for BLPI and WLPI across the pH range tested, with slight differences. Foaming properties were also found to be very similar, and both LPIs had good foam stability at concentrations greater than 1% protein. Major differences were apparent in heat gelling performance; WLPI was able to form a gel at a lower concentration than BLPI. Furthermore, WLPI resulted in stronger gels than BLPI at each concentration, although all gels formed could be described as relatively soft. AA profile of BLPI and WLPI was similar overall; SAAs were the limiting AAs for both ingredients, although their content was slightly higher in BLPI. Both LPIs showed a similar pepsin digestibility and short-term IVPD. While WLPI exhibited significantly reduced TIA levels compared to BLPI, values for both were considered very low. FODMAP content was extremely low for both LPIs, making them suitable for IBS sufferers. Thus, from a nutritional perspective, it can be concluded that LPIs from the blue and white varieties included in this study are very similar. LPIs produced in this way show good nutritional potential, although blending with other proteins may be necessary if a more complete AA profile is desired. The environmental impacts of both LPIs were also very similar, and were shown to be lower than that of cow’s milk protein in all categories except land use, demonstrating that lupin may provide a sustainable alternative to animal protein sources, e.g., dairy. With regard to land use, it is hoped that further development of lupin as a protein crop will result in higher yields leading to improvements in this category. In addition, increase of economic value of lupin processing sidestreams (lupin oil and fibre) would also support reduction of land use per LPI. Overall, protein isolate functionality, particularly with regard to heat gelation, seems be the most important difference between the 2 types of lupin studied here, since it will likely influence which food applications are suitable. 

## Figures and Tables

**Table 1:** Macronutrient and mineral composition of blue lupin protein isolate (BLPI) and white lupin protein isolate (WLPI).

		BLPI	WLPI
Macronutrients	(g/100 g)		
Protein *		92.6 (84.5)	94.4 (86.1)
Fat		0.92	1.07
SFA		0.48	0.65
MUFA		0.25	0.43
PUFA		0.2	<0.10
TFA		<0.10	<0.10
Ash		5.3	4.4
Moisture		5.52	3.81
Dietary fibre		<0.1	<0.1
Minerals	(mg/kg)		
Sodium		14,600	11,740
Potassium		290	320
Phosphorous		9400	8200
Iron		110	60
Zinc		25	22
Copper		11	8
Calcium		1400	1300
Iodine		<10	<10
Magnesium		210	220
Chromium		<1	<1
Manganese		17	170
Molybdenum		2	1
Selenium		1	1
Chlorine		<5	<5

* Protein content is shown as N*6.25, and as N*5.7 in parentheses for comparison. SFA: saturated fatty acids; MUFA: mono-unsaturated fatty acids; PUFA: poly-unsaturated fatty acids; TFA: trans fatty acids.

**Table 2:** Particle size parameters and surface hydrophobicity for BLPI and WLPI.

	BLPI	WLPI
Particle size distribution (μm)		
D_4,3_	12.1 ± 1.20	51.5 ± 6.36
D_3,2_	8.37 ± 1.68	32.7 ± 5.55
Dv (10)	4.76 ± 1.23	19.6 ± 5.46
Dv (50)	9.49 ± 1.79	49.0 ± 5.83
Dv (90)	18.5 ± 1.22	86.6 ± 9.66
Surface hydrophobicity (−)	2185 ± 67.0	842 ± 274

D_4,3_: volume-weighted mean particle diameter; D_3,2_: surface-area weighted mean particle diameter; Dv (10): 10th volume percentile; Dv (50): 50th volume percentile Dv (90): 90th volume percentile. Results are presented as mean ± standard deviation. BLPI and WLPI were significantly different (*p* < 0.05) for all parameters tested.

**Table 3:** Amino acid profiles for BLPI and WLPI, with results expressed as g/100 g protein (N*6.25). Additionally, shown are the levels of indispensable/conditionally indispensable amino acids in BLPI and WLPI as a percentage of the World Health Organisation (2007) adult requirement for each amino acid [75].

	BLPI		WLPI	
	Level (g/100 g Protein)	% of Requirement	Level (g/100 g Protein) ^1^	% of Requirement
Indispensable/conditionally indispensable amino acids				
Histidine	2.53 ± 0.31	168	2.09 ± 0.25	139
Isoleucine	3.97 ± 0.48	132	4.49 ± 0.54	150
Leucine	6.86 ± 0.83	116	7.62 ± 0.92	129
Lysine	4.19 ± 0.51	93	4.25 ± 0.52	95
Methionine	0.33 ± 0.02		0.26 ± 0.02	
Cysteine	1.13 ± 0.08		1.10 ± 0.08	
Methionine + cysteine	1.46 ± 0.11	66	1.36 ± 0.10	62
Phenylalanine	3.88 ± 0.47		4.60 ± 0.56	
Tyrosine	3.01 ± 0.37		5.23 ± 0.63	
Phenylalanine + tyrosine	6.90 ± 0.84	182	9.83 ± 1.19	259
Threonine	3.29 ± 0.4	143	3.30 ± 0.4	144
Tryptophan	0.67 ± 0.1	112	0.57 ± 0.1	95
Valine	3.46 ± 0.42	89	3.65 ± 0.44	94
Dispensable amino acids				
Aspartic acid	9.59 ± 1.16		10.81 ± 1.31	
Glutamic acid	22.77 ± 2.76		23.13 ± 2.8	
Alanine	2.93 ± 0.36		2.94 ± 0.36	
Arginine	11.71 ± 1.42		11.60 ± 1.41	
Glycine	3.86 ± 0.47		3.79 ± 0.46	
Proline	4.41 ± 0.53		3.97 ± 0.48	
Serine	4.88 ± 0.59		5.57 ± 0.67	

**Table 4:** In vitro protein digestibility (IVPD) ^1^ and trypsin inhibitor activity (TIA) ^2^ of BLPI and WLPI.

	IVPD (%)		TIA	
	Pepsin	Pepsin + Pancreatin		
	1 h	Short-Term 1 + 1 h	TIU^3^/mg Sample DM^4^	TIU/mg Protein
BLPI	3.2 ± 0.4 ^a^	36.8 ± 1.2 ^a^	0.12 ± 0.01 ^a^	0.14 ± 0.01 ^a^
WLPI	3.7 ± 0.7 ^a^	35.7 ± 2.0 ^a^	0.09 ± 0.01 ^b^	0.11 ± 0.01 ^b^

^1^ IVPD (%) following pepsin digestion (1 h) or short-term pepsin + pancreatin overall protein digestion (1 + 1 h). IVPD results are presented as mean ± standard deviation. Values followed by different letters are significantly different (*p* < 0.05). ^2^ TIA levels (TIU/mg DM) are based on sample mass or protein mass on dry weight basis, and expressed as TIU/mg sample DM or TIU/mg protein DM, respectively. TIA results are presented as mean ± SD (*n* = 3) for BLPI and mean ± SD (*n* = 2) for WLPI. Values within each column followed by different letters are significantly different (*p* < 0.05). ^3^ TIU: trypsin inhibitor units. ^4^ DM: dry matter.

**Table 5:** Fermentable oligo-, di- and monosaccharides, and polyols (FODMAP) content of BLPI and WLPI.

(g/100g DM) ^a^		BLPI	WLPI *
Mono-/Disaccharides ^b,c^	Glucose	0.06 ± 0	0.01 ± 0
	Fructose	0.08 ± 0	0.01 ± 0
	Excess Fructose ^d^	0.03	−
Polyols ^b^	Xylitol	n.d.	n.d.
	Sorbitol	n.d.	n.d.
	Mannitol	n.d.	n.d.
	∑Polyols	n.d.	n.d.
Oligosaccharides	Raffinose/Stachyose ^b^	n.d.	n.d.
	Verbascose ^b^	n.d.	n.d.
	∑GOS	n.d.	n.d.
	Total fructan ^e^	n.d.	n.d.

^a^ extractions carried out in duplicate and measured via high performance anion-exchange chromatography coupled with pulsed amperometric detection (HPAEC-PAD), results referred to dry matter (DM). ^b^ n.d., not detected or levels below 0.005 g/100 g DM. ^c^ no lactose detected in the ingredients. ^d^ Excess fructose = fructose-glucose. ^e^ n.d., not detected in means of no significant difference in sucrose values and fructose values determined from difference of assay A and B in fructan determination, or levels below 0.1 g/100 g DM. * data as reported by Ispiryan et al. [39].

**Table 6:** Environmental impact profile of BLPI and WLPI per kg protein.

	BLPI	WLPI
Environmental impact potentials (Life Cycle Assessment):		
Climate Change (kg CO_2_-e/kg protein)	6.58	6.40
Aquatic Eutrophication (g PO_4_-e/kg protein)	40.4	39.6
Terrestrial Eutrophication (g PO_4_-e/kg protein)	2.42	2.33
Acidification (g SO_2_-e/kg protein)	33.3	32.5
Photochemical Oxidant Formation (g O_3_-e/kg protein)	12.9	12.6
Fine Particulate Matter (g PM2.5-e/kg protein)	27.5	26.8
Stratospheric Ozone Depletion (mg CFC11-e/kg protein)	37.5	35.4
Additional indicators at the inventory level (LCI):		
Phosphorus Use (g/kg protein)	541	531
Cumulative Energy Demand, non-renewable (MJ/kg protein)	99.9	97.4
Blue Water (process) (kg/kg protein)	140	137
Land Use (m^2^/kg protein)	37.5	36.8
